# The effectiveness of cognitive-behavioral group therapy training on improving emotional intelligence and general health of adolescents

**Published:** 2015

**Authors:** M Aghel Masjedi, M Taghavizadeh, N Azadi, F Hosseinzadeh, A Koushkestani

**Affiliations:** * Clinical Psychology, Islamic Azad University, Tonekabon Branch, Iran; ** General Psychology, Islamic Azad University, Roudehen Branch, Iran; *** Clinical Psychology, Islamic Azad University, Science and Research Branch, Zanjan, Iran; **** General Psychology, Islamic Azad University, Science and Research Branch, Tehran, Iran; ***** Social Sciences Research, Islamic Azad University, Science and Research Branch, Tehran, Iran

**Keywords:** cognitive-behavioral group therapy training, emotional intelligence, general health, male adolescents

## Abstract

**Introduction:** The aim of the current research was to examine the effectiveness of cognitive-behavioral group therapy (CBT) training on the general health and improve the emotional intelligence of male adolescents in Tehran city.

**Methodology:** The current research is a semi-trial research with pretest-posttest experimental design and two test and control groups, which were carried out in the 2014-2015 academic year. 40 high school male students were chosen via proper sampling approach and they were stochastically classified into test and control team (each team, n = 20). The students were protested via Baron emotional intelligence and GHQ-28 general health questionnaire. Subsequently, the test group was trained in the cognitive-behavioral group therapy for eight sessions and the control group received no interventions. In the end, both groups were post-tested, and the data were investigated by using a multivariate investigation of covariance method and SPSS-20.

**Findings:** The outcomes demonstrated that there were notable distinctions between the experiment and the checking teams after the implementation of the CBT training (P < 0.001) so that the average score of emotional intelligence and general health increased in test group rather than in the check team.

**Conclusion:** The findings indicated that the CBT practice is useful in improving emotional intelligence and general health in adolescent male students. Thus, one can recommend that appropriate therapy training could be designed to improve their emotional intelligence and general health.

## Introduction

Scientists, psychologists, and authors have always given a considerable attention to adolescence due to its role and importance in human life [**9**]. This period has also been called emotivism period, emotionalism period, constructive crisis period, and pressure and storm period by various psychologists [**12**]. All these references and explanations showed the importance of this period in human life. Moreover, all these features can cause perturbations, anxieties and changes in an adolescent and also unusual looks may be revealing in his/ her appearance [**9**]. 

In this condition, paying attention to the skills and psychological needs in order to increase the adaptability and proper education of adolescents is of great importance. The emotional intelligence is one of the most crucial skills that helps every adolescent deal with the upward and feathers of his/ her life and helps him/ her not to lose the obtained abilities and virtues with patience [**7**]. Mentally smart means the capability to realize the other people’s emotions and respond to them appropriately; it also means to stimulate, to be aware of, to regulate and to control one’s emotional response [**6**]. The emotional intelligence has a great importance in the period of adolescence. So that Safavi, Mousavi Lotfi and Lotfi (2009) [**10**] believe that one of the most important reasons for adolescents’ conflicts is the psychological disorder, addiction, running away from home, delinquency and so on, originating from the loss of their emotional competencies. In fact, the mechanisms involved in adolescents conflicts that underlie such serious problems, are associated with deficiencies in the individual’s abilities and social or emotional competencies. However, the emotional intelligence can improve at any age through training [**7**]. Consequently, it seems that implementing well-organized strategies aiming the emotional reform can point the adolescents in the right direction. 

Moreover, attention to the adolescents’ health promotion is another crucial factor of providing growth conditions for them. According to the World Health Organization [**14**], health is the entire bodily state, social and emotional well performing. The health status not only depends on one’s body functions, but it also depends on many psychological and social aspects of a person. In the meantime, paying attention to various aspects of adolescents’ health, who are dealing with numerous challenges regarding their period of adolescence, and are passing through one of the most critical stages of their life, is of great importance. In this period, the teenager is physically growing, emotionally immature, with limited experiences and regarding social culture is very fragile and susceptible [**2**]. Therefore, there would be sources of psychological stress in people, and sometimes their health could be in danger, or their adaptability would be affected. In this case, there could be chances for the deviations in some adolescents (Mirzaeemehr, 2014). Consequently, providing their general and psychological health is one of the major tasks of educational and health practitioners. 

One of the therapeutic interventions that has been shown effective is CBT. CBT is a kind of psychotherapy that concentrates on the way people think about a situation. In other words, the people’s emotions are influenced by the way they think, and it assists people know their thoughts, feelings, and attitudes that have an impact on their behavior [**15**]. The CBT is a short-term training and during the training period, which is of 8 to 12 sessions, one can learn how to recognize and change the destructive and disturbing feelings and thinking about patterns that have negative impacts on their behavior [**16**]. There is a strong emphasis in CBT to express the concepts operationally and empirically validate the therapy. Most of the treatment takes place according to the “here and now” technique and it is assumed that the primary goal is to help the patient in a way that he/ she can bring some favorable changes in his/ her life [**4**]. Therefore, during the therapy, people will learn to control their thoughts, how to recognize those thoughts that cause their feelings and actions, and they will also be provided an opportunity for new adaptive learning and making some changes outside the clinical areas (Young, 2007). According to what was said, it can be estimated that the cognitive-behavioral group therapy training would have a significant impact on the improvement of emotional intelligence and general health. Thus, more detailed studies require a better understanding of this subject. However, the literature review showed that no research comprehensively discusses the effectiveness of CBT on improving the emotional intelligence and the general health state in Iran. Therefore, in order to address these research gaps in Iran, and also the need to improve the emotional intelligence and the general health of adolescents, such studies seem to be necessary. Considering the above-mentioned issues, the current research aimed to study the efficacy of CBT training on improving the emotional intelligence and the general health in adolescents. 

## Methodology

The current research was a semi-trial research with a pretest-posttest experimental design and 2 experiment and checking teams. The research sample involved male high school pupils in the first semester of the 2014-2015 academic year. Considering the minimum sample size in experimental researches, which is of 15 people for each group [**13**], 15 people were selected for each cluster. Then, the sample size was considered as 20 people, to increase the statistical power and manage the possible decline in the participants. The samples were selected from all the high schools in Tehran by using the convenience sampling method. Participation criteria for the present study included an informed consent and willingness to participate in the study, willingness to take part in the training sessions, cooperation in completing the tools, having physical and psychological stability, and the age range of 14 to 18 years. Also, a person was excluded from the study if he/ she did not wish to participate. The same happened if he/ she was absent in more than three sessions during the training process, was not able to participate in the sessions, was unwilling to do assignments, had a psychological problem or received other psychological or medical therapies which were not part of this research program. These inclusion and exclusion criteria were considered to control the destructive factors of the results. The tools used in this study included Bar-on emotional intelligence and GHQ-28 general health questionnaire. 

The emotional intelligence questionnaire (EQ-i) which was first used by Bar-on in 1977 and consists of 90 questions whose answers are on a five-point Likert rate, from “I fully agree” (score 1) to “I completely disagree” (score 5). The whole rate of the test was the sum of the 15 scales in which the minimum and maximum score for the participants was considered 90 and 450, respectively. This test has five factors including the combination of interpersonal relationships, intrapersonal relationships, dealing with pressure, adoption, and general mood [**6**]. The reliability of this questionnaire was calculated by Bar-on in seven samples from various populations by using a retest technique within a month. The average of Cronbach’s alpha coefficient was reported in the range of 0.69 (social responsibility) to 0.86 (self-esteem) for each subscale, and the average was 0.76. The evaluation of reliability by using retest showed that the average reliability factor is 0.66. The reliability of the questionnaire was estimated in Iran by Samuel et al., who used the internal consistency method with a Cronbach’s alpha equal to 0.93, being 0.88 while using the even-odd method. In the current study, the confirmation factor was estimated at 0.81 by using Cronbach’s alpha.

General health questionnaire (GHQ) is one of the most commonly used screening devices in psychology and evaluation of mental health conditions and has different types of a questionnaires including 12, 20, 28, 30 and 60 questions. The questionnaire with 28 questions made by Goldberg and Hiller is based on the original questionnaire, and it consists of 4 subscales involving anxiety, physical symptoms, depression and social dysfunction, in which each one had seven questions. This questionnaire has a total score, in which the options are scaled from 0 to 3. The maximum and minimum rates in each scale are 21 and 0, respectively, the total score ranges from 0 to 84 on four scales, and the evaluation is based on obtained scores. Therefore, if the derived score is in the range of 0-21, it shows that the person is in an excellent condition regarding the general health. If the obtained score is in the range of 22-42, it indicates that the individual’s general health was threatened in some cases. If the obtained score is in the range of 43-63 it suggests that the person’s general health was threatened in many cases, and he/ she should think about improving his/ her living condition and general health. Moreover, if the obtained score is in the range of 64-84, it reveals that the person’s general health is in a critical condition, and he/ she should visit a specialist. The reliability and validity of this questionnaire are reported to be 80% in various studies [**8**]. In another study, Yaghoubi et al. determined the sensitivity, specificity, and reliability of this questionnaire on the free population in the best cutoff point 23, which was 0.86, 0.82, and 0.88, respectively.

The research method consisted of the following steps: first, some high school students were selected by using the convenience sampling method in Tehran. Then, 40 students who had the entry criteria assigned into two groups used the randomized block method. Subsequently, one group was randomly considered the test group and the other one was considered the control group. Next, Bar-on emotional intelligence and GHQ-28 general health questionnaire were performed as the pretest on each group and the data were collected. In the next step, the test team was trained in eight sessions of CBT. The sessions were held on a weekly basis, and each one lasted for 90 minutes. 

The educational content of the sessions included the way they would be tested, performing relaxation exercises, meditation, and contemplation and teaching them to perform these exercises every day, teaching self-awareness methods, and designed open-answer questionnaires, at the end the participants being post-tested and the data being collected. The CBT protocol is presented in **Table 1**. 

At the end, in order to analyze the data, descriptive statistical indexes such as average, nominal deviation, frequency, and inferential analytical indexes such as multivariate analysis of covariance by using SPSS-20 software were used. 

**Table 1:** Protocol of problem-solving training sessions based on Dezorila and Glad Frid’s pattern

Sessions	Subject
First	Introducing the people in the group, introducing the group policies, introducing depression, anxiety and stress and awareness of their physical impacts
Second	Recognizing negative thoughts, how these thoughts develop, learning how to overcome the negative thoughts
Third	Learning to overcome the dichotomous thoughts, arbitrary perceptions, unbalanced judgment, instant conclusion, mind reading, misconceptions
Fourth	Learning to overcome the extreme generalization, labeling, inexact terms, exaggerated generalization, absolutism, mental filter and feeling guilty
Fifth	Learning to overcome grandiosity and lowliness, being a tragedy, being disastrous, two part juggling, excessive attention to negative situations, personalization
Sixth	Knowing when you are becoming angry, control and overcome the anger
Seventh	Continuing learning, performing exercises, learning relaxation techniques to use in distressing situations
Eighth	A brief review on training sessions and giving feedback, learning to transfer results to the outside of the group

## Results

The average and the nominal deviation of ages of participants in the current study was 15.73 ± 0.94, and all the participants were male adolescents. 

The descriptive statistics regarding the mean and nominal deviation of feeling smart and general health in both groups is presented in the pretest and posttest stage in **Table 2**. 

**Table 2:** The descriptive statistics regarding emotional intelligence and the general health of both groups for the pretest and posttest stage

variable\group	Number	Index	Emotional intelligence		General health	
			Pretest	Posttest	Pretest	Posttest
Test	15	Mean	226.50	250.75	43.01	54.15
		S.D.	(10.58)	(13.75)	(5.80)	(8.54)
Control	15	Mean	229.15	230.17	42.91	43.01
		S.D.	(13.45)	(11.94)	(5.21)	(5.33)

As it was presented in **Table 2**, the emotional intelligence scores of the test team have risen in the posttest stage rather than in the pre-test stage. However, the average scores in the control team were a little different. The general health scores for the test group have witnessed a rise in the posttest stage rather than in the pretest stage, despite the slight distinction between the scores in the checking team. Since covariance multivariate analysis was used to investigate the information, the default equality of variances (The Levene’s test) was examined, and the results were provided in **Table 3**. 

**Table 3:** The results of Levene’s test which examined the default equality of the variances in the posttest stage

Variable	Stage	F	DOF 1	DOF 2	Significance stage
Emotional intelligence	Posttest	0.189	1	28	0.667
General health	Posttest	2.967	1	38	0.093

As it could be observed from the table above, the default equality of variances was confirmed in both stages. **Table 4** represents the findings of covariance multivariate investigation on posttest scores with a pretest control in emotional intelligence and general health variables. 

**Table 4:** The results of Levene’s test which examined the default equality of the variances in the posttest stage

The test	Value	F	Degree of freedom	Significance level	η^2	Power
Pillai’s Trace	0.504	18.831	2	0.001	0.504	0.95
Wilks Lambda	0.496	18.831	2	0.001	0.504	0.95
Hotelling’s Trace	1.018	18.831	2	0.001	0.504	0.95
Roy’s Largest Root	1.018	18.831	2	0.001	0.504	0.95
p ≤ 0.001						

As it was presented in **Table 4**, the significance leveling of all the tests (p < 0.001) suggested that there was at least one dependent variable (emotional intelligence and general health) that had a clear distinction between the test and the checking team. According to it, 0.50% of the observed difference was related to the independent variable, i.e. the intervention method (cognitive-behavioral group therapy). On the other hand, since the statistical power was 0.95 and it was more than 0.80, the sample size was acceptable for this research. The results related to the significant difference in each independent variable are listed below. 

**Table 5:** The findings of univariate investigation of covariance from the study of the efficacy of training the spiritual intelligence components on the general and emotional health in posttest stage

Index	Sum of squares	DOF	Average square	F	Significance leveling	η^2
Emotional intelligence	4243.601	1	4243.601	26.365	0.001	0.411
General health	1243.225	1	1243.225	24.522	0.001	0.392

According to **Table 5**, since the significance leveling was less than 0.05, the theory of difference between emotional intelligence and general health in the posttest stage between the two groups was verified. In other words, 0.41% of the change in the emotional intelligence score of people was because of the change in the independent variable. Thus, the cognitive-behavioral group therapy could improve the emotional intelligence of adolescents. Also, a 0.39% change in the general health score of people was because of the change in the independent variable. Consequently, the cognitive-behavioral group therapy could cause an improvement in the general health of adolescents. 

## Conclusion

According to the aim of the current research, which was the investigation of the efficacy of CBT regarding the improvement of the emotional intelligence and the general health in adolescents, the results of the covariance multivariate investigation indicated that the CBT had a clear impact on the improvement of the emotional intelligence and the general health in adolescents. These findings were consistent with various studies such as Shaghaghi (2010) and Ghamari, Zahedbabolan and Fathi (2005) [**11**]. In the explanation of the same finding, Shaghaghi (2010) suggested that since the attitudes are closely placed in our mental structure, they are related to the more fundamental values and reflect our beliefs about different issues. In addition, if they are evaluated carefully, and other factors such as social norms are considered, they can be some strong estimates of emotions and behaviors, providing the foundation of knowledge in the interactions with others (Mitchell, 1977; quoted from Shaghaghi, 2010). Consequently, one of the most important ways to change the emotion and behavior of individuals is to make a change in their attitudes. Automatic thoughts, which are enabled with different events, are among the most important attitudes related to feelings. Thus, changing these ideas can lead to the recognition and development of emotions. 

The finding that the CBT has a significant impact on the improvement of the general health of adolescents is inconsistent with the study of Rajabi and Karjou Kasmaee (2012) [**5**], Ahadi, Yousefi Louyeh, Salehi and Ahmadi (2009) [**1**] and Khodayari, Abedini, Akbari, Ghbari Bonab, Sohrabi and Younesi (2008) [**3**]. In the explanation of the same finding, Rajabi and Karjou Kasmaee (2012) [**5**] suggested that the CBT could form and organize the individual’s perceptions of events such as health or sickness. Not only could it have an impact on the person’s health, but also it could be useful in the life’s harsh experiences such as sadness or loss, being also effective in other aspects of a person’s well-being. In addition, it is said that changing thoughts could help emotional development, adoption, and flexibility through the experience of positive feelings and attention to bright sides of life. Moreover, people’s thoughts are directly related to positive expressions and perception of health. Ahadi, Yousefi Louyeh Salehi and Ahmadi (2009) [**1**] also suggested that there is a relation between thoughts and health. However, this relationship is very complicated and has a very beneficial effect on the physical health and psychological attitudes. The recent studies showed that changing thoughts are related to positive outcomes such as physical, emotional, and psychological well-being, positive intrapersonal interaction, marital satisfaction and stability and improvement of the quality of life. Having a balanced attitude regarding life may be a contributing factor in these positive outcomes, which prevent inconsistent and undesirable behaviors such as acting in personal and social destructive ways. Furthermore, it was found that the tendency towards the fundamental concepts of life could lead to the experience of being purposeful, having satisfaction, and acquiring personal integrity. 

**Acknowledgement**

The authors would like to thank authorities in the minister of education in Tehran province for their contribution to the study. They would also like to thank all the participants in this study.
